# Epigenetic aging clocks in mice and men

**DOI:** 10.1186/s13059-017-1245-8

**Published:** 2017-06-14

**Authors:** Wolfgang Wagner

**Affiliations:** 1 Helmholtz-Institute for Biomedical Engineering, Stem Cell Biology and Cellular Engineering, University Hospital of the RWTH Aachen, 52074 Aachen, Germany; 2 Institute for Biomedical Engineering — Cell Biology, University Hospital of the RWTH Aachen, 52074 Aachen, Germany

## Abstract

Epigenetic clocks provide powerful tools to evaluate nutritional, hormonal, and genetic effects on aging. What can we learn from differences between species in how these clocks tick?

Please see related Research articles: http://genomebiology.biomedcentral.com/articles/10.1186/s13059-017-1203-5, http://genomebiology.biomedcentral.com/articles/10.1186/s13059-017-1186-2, http://genomebiology.biomedcentral.com/articles/10.1186/s13059-017-1187-1 and http://genomebiology.biomedcentral.com/articles/10.1186/s13059-017-1185-3

One of the most fascinating findings in human aging is that it is associated with highly reproducible DNA methylation (DNAm) changes [1]. DNAm levels at age-associated CG dinucleotides (CpG sites) can be integrated into epigenetic age predictors, which provide robust biomarkers to estimate chronological age. With the advent of more and more publically available DNAm profiles, such aging signatures were further developed to facilitate higher precision in age predictions, particularly for blood samples [2, 3]. Probably the most commonly used epigenetic aging signature has been described by Horvath [4]. It is based on DNAm levels at 353 CpG sites and facilitates relatively precise age predictions for many human tissues: the median “error” (MAE), defined by the median absolute difference between DNAm age and chronological age, is usually less than 4 years.

Now—about 6 years after the first epigenetic clock paper—similar age predictors have been established for mice [5–7]. Again, they were initially described for defined murine tissues, specifically liver by Wang et al. [5] and blood by Petkovich et al. [6], taking into account the fact that there are notoriously large differences in the epigenetic makeup of cells from different tissues. However, Stubbs and coworkers have demonstrated that it is also possible to derive a multi-tissue murine DNAm age predictor [7], in analogy to the Horvath clock. Their signature is based on 329 CpGs and has been validated for cortex, muscle, lung, liver, and heart tissue [7]. Overall, the multi-tissue age predictor reached a MAE of less than 4 weeks, although how it performs in other tissues has yet to be shown.

## Differences between human and mouse clocks

All three studies mentioned above indicate that the epigenetic clocks of mice tick faster than those of humans. This can be anticipated because the maximum life-span of mice (about 2 years) is much shorter than it is in humans (about 85 years). If the molecular changes of aging are linked to life expectancy and generation time, then this might support the notion that aging reflects a controlled evolutionary process. However, there is still an open debate on whether aging is due to an accumulation of cellular defects, or is driven by a developmental mechanism. Either way, comparison of epigenetic clocks in mice and men will provide new insights into the regulation of age-associated DNAm (Table 1).

**Table 1:** Comparison of epigenetic aging clocks in mice and men

Feature^a^	Human	Mouse
DNAm datasets used to derive predictors	Microarray data	RRBS and WGBS
Signatures for specific tissues	Blood [2, 3]	Liver [5] and blood [6]
Multi-tissue age predictor	Horvath predictor [4]	Stubbs et al. predictor [7]
MAE of multi-tissue age predictors	Usually <4 years (~5% of lifespan)	3.33 weeks in test dataset (~5% of lifespan)
Predictors based on individual CpGs	Multiple assays described (for example, [3])	Not yet described
Association of DNAm age with gender	Female samples are predicted to be younger [3, 4, 12]	No evidence so far [7]
Association of DNAm age with life expectancy	Yes—with higher all-cause mortality [11]	Yes—evidence from long-lived mice [5]
Association of DNAm age with nutrition	Accelerated epigenetic age in higher BMI [13]	Caloric restriction significantly delays epigenetic aging [5, 6, 9, 10]
Clock reset on reprogramming into iPSCs	Yes [3, 4]	Yes [6]

^a^Note that few studies in mice have been carried out so far, so the information is based on a small number of studies published at the time of writing. *DNAm* DNA methylation, *iPSC* induced pluripotent stem cell, *MAE* median absolute error, *RRBS* reduced representation bisulfite sequencing, *WGBS* whole-genome bisulfite sequencing

Direct comparison of age-associated CpGs in mice and men indicated that there is a moderate but significant association between the two species [5, 6]. It is not always trivial to identify orthologous CpG sites, and further interspecies comparison will be required to better understand similarities and differences of age-associated genomic regions. However, the overlap of age-associated CpGs in age predictors for human and mice seems to be rather low [5, 7], and hence epigenetic clocks need to be trained specifically for different species. There may even be some relevant differences in the epigenetic clocks of different mouse strains, although so far this was not evident [5, 7].

In terms of function, age-associated CpGs in humans and mice seem to be enriched in genes that are involved in morphogenesis and development [3, 7, 8]. However, in both species age-associated DNAm changes are not generally reflected at the gene expression level—and thus the biological relevance remains largely unclear. Another recent study suggests that only a specific subset of differentially methylated regions (DMRs) is related to transcriptional and functional outcomes in aging mice [9].

## Regulation of age-associated DNAm patterns

How are age-associated DNAm patterns regulated at the molecular level? Age-associated hypermethylation and hypomethylation follow different patterns in humans and mice, and may therefore be controlled by different molecular processes. In both species, hypermethylation is enriched at CpG islands (CGIs), whereas hypomethylation is rather observed in regions outside of CGIs [10]. Notably, Stubbs and coworkers found that hypermethylation was enriched in the shore and shelf regions of CGIs and in non-CGI promoters [7], indicating that a better understanding of the characteristic features of DMRs is required. It is entirely possible that age-associated DNAm changes reflect other functional changes in chromatin conformation. In fact, age-associated hypermethylation in mice seems to be enriched in genomic regions with bivalent activating and repressing histone marks [10], as previously shown for humans, indicating that there is a link to the dynamic nature of other chromatin modifications. It has been suggested that age-related methylation changes are caused by “epigenetic drift”—a gradual loss of control of DNAm patterns over time. On the other hand, stochastic changes should be acquired at a similar rate in different species. The faster pace of murine epigenetic clocks may therefore indicate that they can be controlled. This is also supported by the notion that reprogramming of adult cells into induced pluripotent stem cells (iPSCs) resets the epigenetic aging clock to close to zero in humans [3, 4] and mice [6]. Hence, it is possible to epigenetically rejuvenate cells by conversion to the pluripotent state.

## Powerful tools

The murine DNAm clocks provide powerful tools to study longevity interventions in one of the most relevant model organisms for aging research. These signatures were initially trained to correlate with the “real” chronological age of mice—but aging rates may differ between individuals. In fact, there is evidence that epigenetic clocks rather reflect the biological age, which is related to the perceived aging process of an organism. In analogy, Marioni et al. [11] previously demonstrated that human DNAm age is related to life expectancy: accelerated epigenetic age is associated with higher all-cause mortality. This finding has been validated in various additional cohorts and with different epigenetic age predictors. Furthermore, human epigenetic aging rates have been shown to be significantly associated with sex, race/ethnicity, and some disease risk factors [12]. In mice, there was no clear difference in predicted DNAm age of male and females [7]. However, ovariectomy, which reduces the average life span in female rats, results also in significant age acceleration [7]. Caloric restriction [5, 6, 9, 10] or dietary rapamycin treatment [5], both of which result in increased life expectancy of mice, reduced epigenetic age. Notably, mice fed with a high-fat diet showed accelerated epigenetic aging, which had a tendency to be further exacerbated if the mothers were fed a low-fat diet [7]—thus, there may even be trans-generational effects on epigenetic age. In humans, specific diet seems to have a less pronounced impact on epigenetic age, but there is significant association of DNAm age and body mass index (BMI) [13]. Apparently, different parameters can affect biological aging in mice and men.

## The path ahead

The main reason why epigenetic aging clocks in mice were described several years after those in humans is a simple technical issue. The epigenetic aging clocks in humans were exclusively based on Illumina Bead Chip microarray datasets. These platforms facilitate profiling of DNAm levels at about 27,000 CpGs (27 k BeadChip), 450,000 CpGs (450 k BeadChip), or more than 850,000 CpGs (EPIC BeadChip) at single-nucleotide resolution. As these microarrays have been widely used, human DNAm profiles can be easily retrieved from public data repositories for cross-comparison of the same CpGs.

However, such microarrays are not available for mice. Therefore, DNAm clocks for mice had to be established based on datasets that were either generated by reduced representation bisulfite sequencing (RRBS) or whole-genome bisulfite sequencing (WGBS). Particularly in the case of RRBS, not all CpG sites are covered in all samples and a limited number of reads may entail lower precision of DNAm level measurements at individual CpGs. In the study by Stubbs and colleagues, 730,000 CpG sites had more than fivefold coverage in all samples analyzed—despite sequencing to 15× genomic coverage on average [7]. It may, therefore, not be trivial to apply the murine DNAm clocks to other datasets, which are known to be missing some of the relevant CpGs.

The wide use of Horvath’s clock is at least partly based on the ease of applicability for other researchers. He has provided a detailed R software tutorial as well as a user-friendly web implementation [4]. Further development of the pipelines for RRBS-based DNAm clocks will probably not only increase precision, but may also address the technical limitations of coverage and sequence variations—and provide a more user-friendly interface for data processing.

Intervention studies for aging research usually necessitate many biological replicas; however, studies based on RRBS and—even more so—WGBS are complex and costly. For future research, it would therefore be useful to develop DNAm clocks for mice that are based on site-specific analysis of only one or a few selected age-associated CpGs. For human tissues, multiple studies have described pyrosequencing and MassARRAY assays to determine site-specific DNAm levels and provide relatively precise age predictions [3]. The use of a smaller number of CpGs is a tradeoff between precision and applicability of the method—and hence the “error” with regard to chronological age is usually slightly higher than using signatures based on genome-wide DNAm profiles. It remains to be demonstrated whether site-specific analyses of age-associated CpGs, which can now be identified based on the recent studies, can also facilitate precise estimation of chronological age in mice; and if such simplified measures would also be capable of detecting effects of longevity interventions.

Taken together, the multi-tissue DNAm age predictor for mice provides a new and powerful tool for aging research. Without doubt the DNAm aging clocks will be further developed based on the rapidly growing number of available DNAm profiles and advances in bioinformatics. Relevant parameters for aging research can be better controlled in mice than men, but it needs to be taken into consideration that treatments or genetic modifications may exert different impacts on the epigenetic clocks of the two species. Therefore, a better understanding and inter-species comparison of age-associated DNAm is important and it may even shed light on the underlying molecular process that drives the epigenetic aging clocks—and possibly aging of the organisms.

## Abbreviations

BMI: Body mass index
CGI: CpG island
DMR: Differentially methylated region
DNAm: DNA methylation
iPSC: Induced pluripotent stem cell
MAE: Median absolute error
RRBS: Reduced representation bisulfite sequencing
WGBS: Whole-genome bisulfite sequencing
